# Imaging modalities in early cardiac transthyretin amyloidosis: who is first?

**DOI:** 10.1007/s00392-022-02063-6

**Published:** 2022-07-19

**Authors:** Svenja Ney, Lenhard Pennig, Michael Faßbach, Christopher Hohmann, Roman Pfister

**Affiliations:** 1 Department of Cardiology, Heart Centre, Faculty of Medicine and University Hospital of Cologne, University of Cologne, Kerpener Str. 62, 50937 Cologne, Germany; 2 Institute for Diagnostic and Interventional Radiology, Faculty of Medicine and University Hospital of Cologne, University of Cologne, Cologne, Germany; 3 Praxis Für Innere Medizin Euskirchen, Euskirchen, Germany

**Fig. 1:**
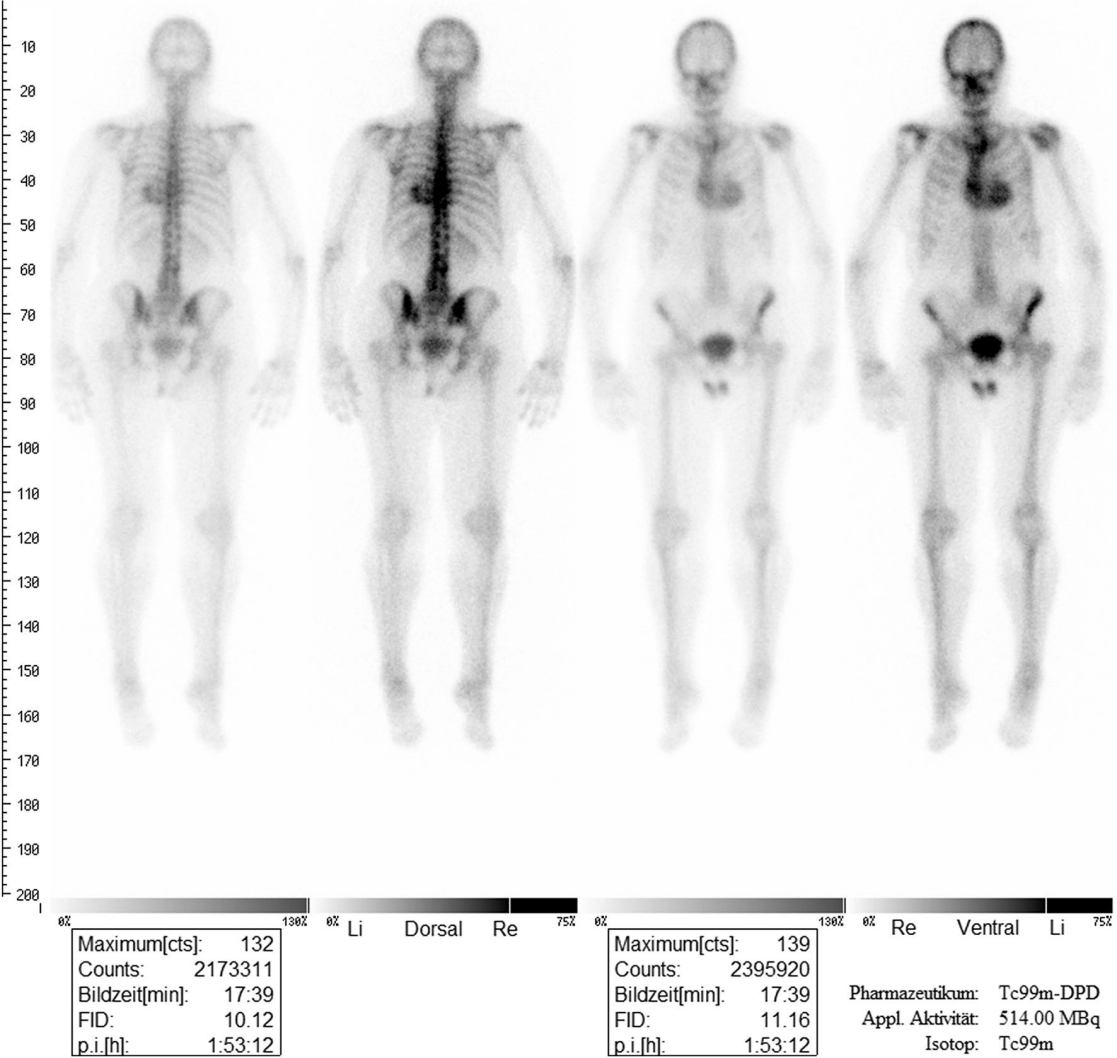
Planar 99mTc-MDP scan reveals Perugini grade 3 myocardial uptake suggestive of cardiac amyloidosis

Sirs:

An 80-year-old woman underwent technetium (^99m^Tc) medronic acid whole body scintigraphy because of arthritis of the right knee joint. Single photon emission computed tomography (SPECT) imaging showed only a minor tracer uptake of the right knee, but a substantial uptake of the heart, corresponding to grade 3 Perugini score (Fig. 1). The patient was transferred to an outpatient cardiologist for suspected cardiac amyloidosis.

**Fig. 2:**
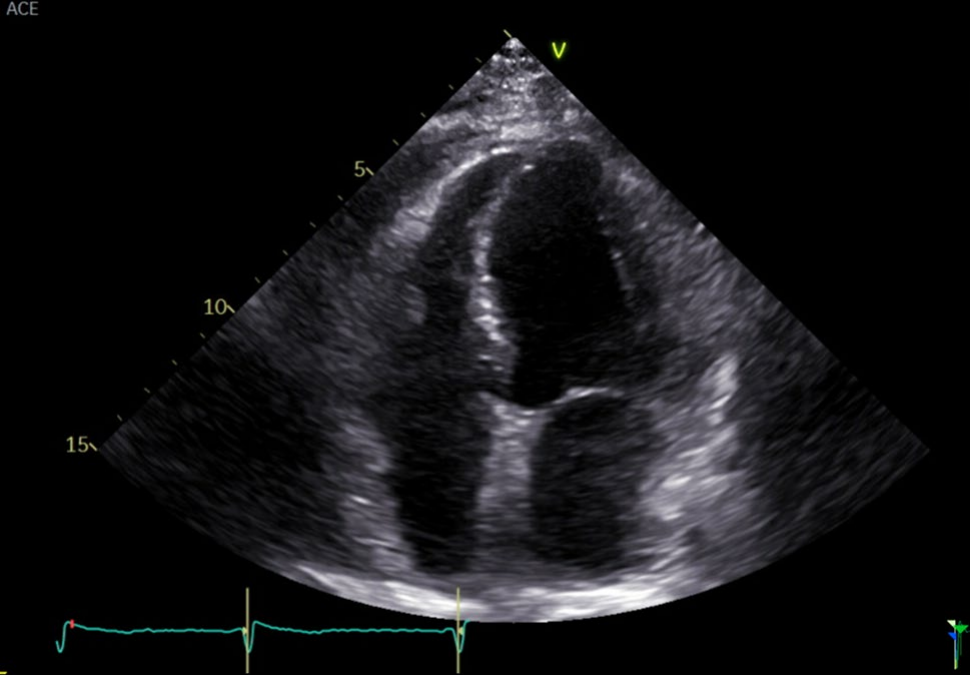
Echocardiography with mild concentric myocardial hypertrophy

**Fig. 3:**
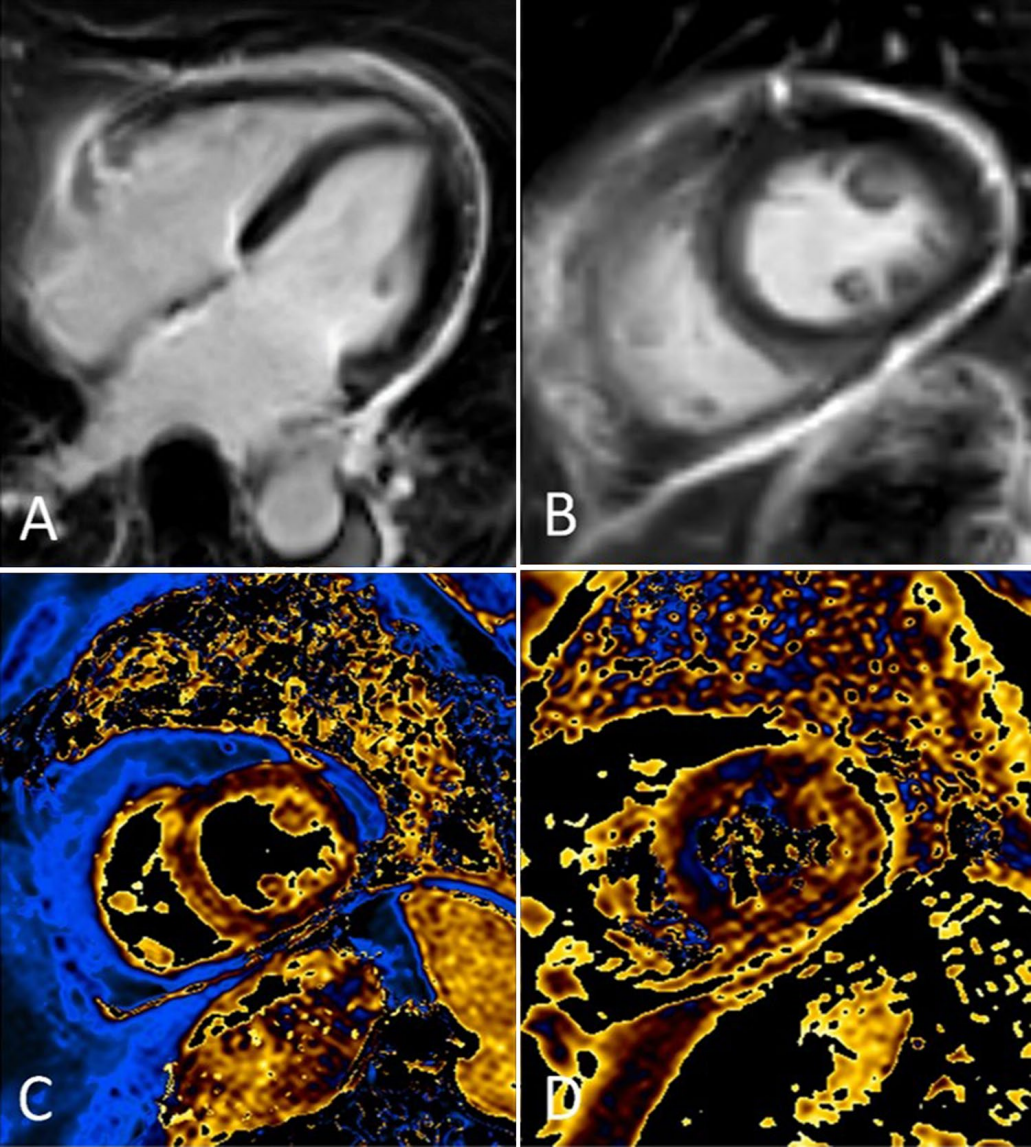
Multiparametric CMR showing a mild concentric hypertrophy of the left ventricle of up to 12 mm in the 4Ch cine (**A**). LGE imaging (**B**, 4Ch) does not reveal an enhancement pattern suggestive of amyloidosis. As depicted in color-coded maps in the short-axis, global T1-(**C**) and T2-(**D**) values were below 1100 and 60 ms, respectively, ruling out a diffuse fibrosis or acute myocardial pathologies. Note the pericardial LGE due to a previous pericardial puncture

The patient had a medical history of Parkinson’s disease, hypertension and bronchial asthma and underwent total endoprosthesis implantation of the shoulder joint in 2016. Blood pressure was well controlled with ramipril 5 mg OD. Physical activity was limited by the orthopedic and neurological diseases, with only mild exertional dyspnea. Physical examination was unremarkable. Resting ECG showed sinus rhythm with low-voltage and regular de- and repolarization. Transthoracic echocardiography (TTE) showed normal heart dimensions with mild, concentric left-ventricular hypertrophy (interventricular septum diameter 12 mm, Fig. 2). Systolic left- (LV) and right (RV) ventricular function were preserved with a mild impairment of global longitudinal strain (− 15%) without a distinct regional pattern and grade 1 diastolic dysfunction. NT-proBNP was mildly elevated (176 pg/ml). Due to the inconclusive echocardiographic results with respect to cardiac amyloidosis, the patient was referred for cardiovascular magnetic resonance (CMR). Here, RV and LV function and total myocardial mass were normal with a mild septal hypertrophy (Fig. 3). Tissue characterization showed no myocardial edema, no typical late gadolinium enhancement (LGE) pattern and normal relaxation times in T1 and T2-mapping, thus providing no evidence for cardiac amyloidosis.

**Fig. 4:**
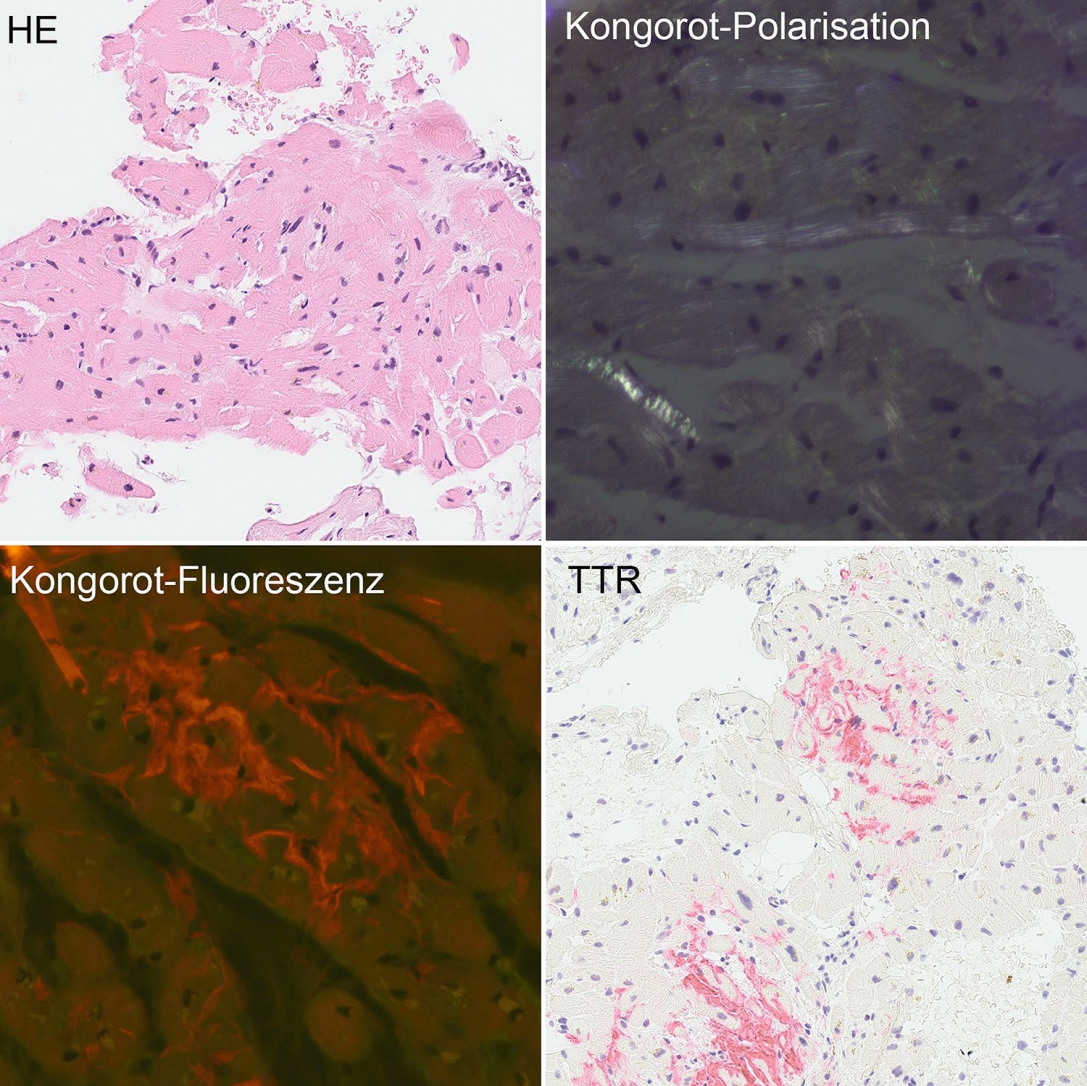
Histological identification of TTR-Amyloid from endomyocardial biopsy

After the still inconclusive results of both TTE and CMR, further non-invasive testing was conducted to evaluate the potential presence of monoclonal protein as an indicator of light-chain amyloidosis (AL). Serum immunofixation electrophoresis demonstrated a distinct IgG-kappa peak. Free serum kappa light chains were mildly increased (43 mg/l, reference 3–19 mg/l), and kappa-lambda ratio was elevated 2, 5 (reference 0.26–1.65). Hence, AL could not be excluded but was not highly probable given the low levels of free light chains. To prove and subtype potential extracardiac amyloidosis, subcutaneous fat biopsy was performed which revealed no evidence of amyloid deposition. Finally, LV myocardial biopsy was conducted, which revealed interstitial amyloid deposition of transthyretin subtype (ATTR, Fig. 4). Given NYHA class II heart failure symptoms, the increased NT-proBNP level and mild morphological abnormalities of the LV as signs of ATTR cardiomyopathy, treatment with tafamidis was initiated.

We present a patient with cardiac ATTR amyloidosis at a very early disease stage incidentally detected by bone scintigraphy with yet inconclusive results of echocardiography and CMR. This case highlights the current lack of information regarding subclinical ATTR manifestations and transition to early disease stage which might impact both the diagnostic algorithm and initiation of ATTR therapy in such disease stage [1].

The advances in imaging set the stage for non-invasive diagnosis of cardiac amyloidosis which was the premise for a broad identification of patients with ATTR cardiomyopathy in recent years. Indeed, cardiac ATTR amyloidosis can be diagnosed without biopsy in many patients. However, the majority of current evidence on the performance of imaging modalities was derived from patients with advanced disease stage. Typical imaging features of cardiac amyloidosis like pericardial effusion, restrictive filling pattern, remarkable hypertrophic phenotype, apical sparing of the reduction in longitudinal strain, and diffuse late gadolinium enhancement correlate with disease progression and little is known on their discriminatory performance in subclinical disease stages.

With the introduction of the first disease modifying therapy of ATTR cardiomyopathy there is a major clinical urge to detect patients early in the disease. The main effect of tafamidis is the prevention of disease progression and hence the therapeutic benefit is expected highest in early disease stages [2]. Importantly though, the evidence from the current phase III trial was derived from patients with clear morphological signs of cardiac amyloidosis and markedly elevated NT-proBNP.

Bone scintigraphy is known to be particular sensitive to detect amyloid deposits and would be first choice for screening of ATTR cardiomyopathy [3]. In line with this, in our patient only scintigraphy and neither echocardiography nor cardiac CMR provided evidence of cardiac ATTR deposits. However, the lack of definitions for very early and subclinical stage of ATTR cardiomyopathy rises the important questions on whether such findings already equate with the diagnosis of ATTR cardiomyopathy and whether therapy should be initiated. According to a current multi-societal expert consensus statement non-invasive diagnosis of cardiac ATTR amyloidosis with bone scintigraphy requires typical imaging features in TTE and/or CMR [4]. In consequence, this means that non-invasive diagnosis of ATTR cardiomyopathy can only be made at advanced stages or biopsy is required to confirm the diagnosis at early stages. Both is counterintuitive for the concept of easy and early detection of ATTR amyloidosis proposed as the key condition for effective therapy and underlines the need of evidence to define early stages of ATTR cardiomyopathy particularly with respect to initiation of medical therapy.
